# Effect of Dietary Capsaicinoids Supplementation on Growth Performance, Intestinal Morphology, and Colon Microbiota in Weaned Piglets

**DOI:** 10.3390/antiox15010129

**Published:** 2026-01-19

**Authors:** Kangwei Hou, Zhixiang Ni, Jiangdi Mao, Haifeng Wang

**Affiliations:** The Key Laboratory of Molecular Animal Nutrition, Ministry of Education, College of Animal Science, Zhejiang University, Hangzhou 310058, China

**Keywords:** capsaicinoids, piglet, growth performance, antioxidation, intestine, microbiota

## Abstract

This study investigated the effects of encapsulated capsaicinoids (CAPs), containing 0.47% capsaicin and 0.22% dihydrocapsaicin, on growth, serum parameters, nutrient digestibility, and intestinal health in weaned piglets. A total of 168 piglets were randomly assigned to four groups: a basal diet or the same diet supplemented with 200 (LDC), 400 (MDC), or 600 (HDC) mg/kg of CAPs. The results indicated that CAPs improved lipid metabolism, evidenced by higher crude fat digestibility in the LDC and MDC groups and reduced serum low-density lipoprotein cholesterol in all CAP groups compared to the control. Glutathione peroxidase activity was significantly higher in the MDC and HDC groups. Histological analysis showed reduced hepatic vacuolation, enlarged fungiform papillae with shallower taste pores in the tongue epithelium, and deeper ileal crypts in the LDC group. At the molecular level, ZO-1 expression in the ileum was significantly upregulated in LDC piglets. Colonic microbiota analysis revealed decreased relative abundances of *Lachnospiraceae_AC2044_group*, *Lachnospiraceae_XPB1014_group*, and *Rikenellaceae_RC9_gut*, while *Butyricicoccus* was significantly enriched in the LDC group. In conclusion, CAPs supplementation enhanced fat digestibility, lipid metabolism, antioxidant capacity, intestinal development, and colonic microbiota composition, with the 200 mg/kg dose showing the most pronounced effects.

## 1. Introduction

Early weaning is a critical management strategy in modern swine production, aimed at shortening the reproductive cycle of sows and improving overall production efficiency. However, the weaning process involves abrupt changes in diet and living environment, which can impose considerable stress on piglets, leading to intestinal dysfunction, immunosuppression, diarrhea, and reduced growth performance [1,2]. Post-weaning mortality can account for over half of total swine farm losses, underscoring the importance of effective nutritional interventions to support piglet health and growth during this critical phase. Among various interventions, antibiotics have been widely used to promote growth and prevent disease due to their efficacy [3]. However, their overuse has raised significant public health concerns, including antimicrobial resistance, drug residues, and environmental pollution, leading many countries to restrict or ban their inclusion in feed and driving the development and application of safe and effective alternatives [4,5].

Plant feed additives have attracted considerable attention due to their natural antimicrobial, anti-inflammatory, and antioxidant properties and are widely used in animal diets to improve growth performance and maintain health [6,7]. Among various plant-derived active substances, chili peppers and their major active constituents, capsaicinoids (CAPs), have become a research focus owing to their anti-inflammatory, antioxidant, and immunomodulatory effects [8]. The main components of CAPs are capsaicin (CAP) and dihydrocapsaicin (DHC), accounting for approximately 80–90% of total content and representing the core of their bioactivity [9,10]. CAPs can activate transient receptor potential vanilloid 1 (TRPV1) channel [11] and modulate inflammation, oxidative stress, and lipid metabolism through both TRPV1-dependent and independent pathways [12]. Furthermore, CAPs have been shown to enhance intestinal barrier function and reshape gut microbiota composition [13,14], highlighting their potential applications in intestinal health regulation. Notably, China is the largest producer and consumer of chili peppers worldwide, with its 2017 production accounting for more than half of global output [15], providing ample resources for the industrial application of CAPs-related products.

However, CAPs are inherently irritant compounds, and excessive supplementation may cause gastrointestinal irritation or toxic effects [16,17], particularly during the stressful weaning period when intestinal burden is already elevated. Moreover, CAPs are sensitive to heat, light, and acidic environments, and direct inclusion in feed can lead to degradation and reduced bioavailability [18]. To overcome these limitations, microencapsulation technology has been employed to process CAPs, allowing delayed release, reducing oral and gastric irritation, and promoting targeted intestinal delivery and absorption, thereby enhancing their physiological efficacy [19,20].

Regarding active ingredient selection, a mixture of CAP and DHC more closely resembles the natural composition of CAPs compared with individual pure compounds. Considering their natural co-occurrence and reported synergistic effects [13], using a combined formulation may achieve superior bioactivity. Therefore, the aim of this study was to systematically evaluate the effects of different doses of an encapsulated CAPs formulation containing 0.47% CAP and 0.22% DHC on growth performance, intestinal health, serum biochemical parameters, nutrient digestibility, and gut microbiota in weaned piglets. We hypothesized that dietary supplementation with encapsulated CAPs would improve intestinal health, antioxidant capacity, and gut microbiota composition in weaned piglets.

## 2. Materials and Methods

### 2.1. Animals and Diets

A total of 168 weaned piglets (21–28 days old, average body weight 7.5 kg) were randomly assigned to 4 groups, each with 7 replicates of 6 piglets, with equal numbers of males and females. The control group was fed the basal diet (CON), while the other three groups received the basal diet supplemented with the encapsulated CAPs formulation at 200 mg/kg (low-dose CAPs, LDC), 400 mg/kg (medium-dose CAPs, MDC), and 600 mg/kg (high-dose CAPs, HDC), respectively. These inclusion levels were selected based on prior studies and practical experience to ensure safe and effective dosing [21]. The encapsulated formulations contained 0.47% CAP and 0.22% DHC and were supplied by Lucta Flavours Co., Ltd. (Guangzhou, Guangdong, China). The basal diet was formulated according to the NRC (2012) recommendations, and its ingredients and nutrient composition are shown in Table 1. The diet included regular soybean meal, expanded soybean, and fermented soybean meal to provide a balanced amino acid profile and improved digestibility for weaned piglets. The feeding trial lasted for 5 weeks. On days 1 and 35, all piglets—following a 12 h fast—were individually weighed at 08:00 to assess growth performance. At the end of the trial, based on the overall growth performance and serum biochemical results which indicated that the low-dose CAPs (LDC) group exhibited the most consistent beneficial responses, twelve male piglets (six each from the CON and LDC groups) with body weights within ±5% of the group mean were humanely euthanized by intraperitoneal injection of sodium pentobarbital, followed by exsanguination via cardiac puncture. Tissues were then collected for in-depth mechanistic analysis.

### 2.2. Growth Performance

Feed intake was recorded every three days. For each growth period of the weaned piglets, the average daily feed intake (ADFI), average daily gain (ADG), and feed-to-gain ratio (F/G) were calculated. ADG was determined by subtracting the initial body weight (IBW) from the final body weight (FBW) and dividing the result by the number of experimental days. The F/G ratio was calculated as ADFI divided by ADG. At the end of the experiment, fecal samples were collected from each group and immediately mixed with 10% hydrochloric acid to prevent nitrogen loss during storage, following the method described by Zuo, et al. [22]. Both diet and fecal samples were finely ground and passed through a 1 mm (40-mesh) screen. The processed samples were then used to determine dry matter (DM; method 930.15), crude protein (CP; method 990.03), and crude fat (CF; method 945.16) following the procedures outlined by the AOAC [23]. Acid-insoluble ash (AIA) was used as an endogenous marker to calculate the apparent total tract digestibility (ATTD) of each nutrient, based on the method described by Liu, et al. [24]. The equation used to calculate ATTD was as follows:

ATTD (%) = 1 − [(AIA diet × Nutrient feces)/(AIA feces × Nutrient diet)] × 100.

### 2.3. Blood Analysis

Before slaughter, blood samples were collected from the jugular vein using vacuum coagulation tubes containing separation gel. The samples were centrifuged at 3000 rpm for 15 min at 4 °C to obtain serum. Commercial assay kits (Nanjing Jiancheng Bioengineering Institute, Nanjing, China) were used to determine serum biochemical and antioxidant parameters. Biochemical measures included triglycerides (TGs), total cholesterol (T-Chol), low-density lipoprotein cholesterol (LDL-C), and high-density lipoprotein cholesterol (HDL-C). Antioxidant assessments comprised total antioxidant capacity (T-AOC), glutathione peroxidase (GSH-Px) activity, superoxide dismutase (SOD) activity, and malondialdehyde (MDA) levels.

### 2.4. Organ Indices

Immediately after slaughter, the heart, liver, spleen, and kidneys were excised and weighed to calculate their respective organ indices. Organ indices were calculated as the ratio of organ weight to body weight.

### 2.5. Morphology Structure Analysis

Samples of the mid-jejunum, mid-ileum, and the left lateral lobe of the liver were collected. The tissues were fixed in 10% paraformaldehyde, embedded in paraffin, and sectioned at 5 µm thickness. The sections were deparaffinized, rehydrated through a graded ethanol series (100%, 95%, and 75%; 15 min each), and stained with hematoxylin and eosin (H&E). Morphological observations were performed using a light microscope (Olympus Corporation, Tokyo, Japan), and villus height and crypt depth were measured using ImageJ software (version 1.52V; NIH, Bethesda, MD, USA). For scanning electron microscopy (SEM) analysis, glutaraldehyde-fixed samples were rinsed three times with phosphate-buffered saline (PBS) for 15 min each, post-fixed in 1% osmium tetroxide for 2 h, and rinsed again three times with PBS. Subsequently, the samples were dehydrated through a graded ethanol series (30%, 50%, 70%, 80%, 90%, and 95%; 15 min each), followed by two washes in 100% ethanol for 20 min each. The dehydrated samples were dried using a Hitachi HCP-2 critical point dryer and observed using a Hitachi SU-8010 scanning electron microscope (Hitachi High-Tech Corporation, Tokyo, Japan).

### 2.6. Western Blot Analysis

Ileal tissue samples were collected, and the expression of tight junction proteins was analyzed using GAPDH as the internal reference, following the method described previously [25]. Proteins were extracted, separated by 10% or 12% SDS-PAGE, and then transferred onto PVDF membranes. The membranes were incubated overnight at 4 °C with primary antibodies against occludin, zonula occludens-1 (ZO-1), and GAPDH (Abcam, Cambridge, UK or CST; dilution 1:1000), followed by incubation with HRP-conjugated goat anti-rabbit IgG secondary antibody (Abbkine, Atlanta, GA, USA; dilution 1:5000). Protein bands were visualized using enhanced chemiluminescence (ECL) reagents (Beyotime, Shanghai, China), captured with a gel imaging system, and quantified using ImageJ software after background correction.

### 2.7. 16SrDNA Sequencing

Immediately after slaughter, colonic contents were collected into sterile centrifuge tubes, flash-frozen in liquid nitrogen, and stored at −80 °C for subsequent analysis. The V3–V4 region of the 16S rRNA gene was amplified, and PCR products were quantified using a Qubit fluorometer (Invitrogen, Waltham, MA, USA). Sequencing was conducted on an Illumina NovaSeq PE250 platform (Illumina, San Diego, CA, USA), followed by bioinformatics analysis. Chimera sequences were identified, and de novo operational taxonomic units (OTUs) were clustered at a 97% sequence identity threshold using Usearch (v7.0) and UPARSE (https://drive5.com/uparse/, accessed on 15 March 2025). QIIME2 (version 2024.2) was employed to analyze bacterial α- and β-diversity at both phylum and genus levels, as well as relative abundance and taxonomic differences across groups. Principal coordinates analysis (PCoA) based on weighted UniFrac distances was used to visualize differences in microbial community structure between groups. In addition, principal component analysis (PCA) was performed on the normalized OTUs abundance data to further assess overall variance and sample clustering. Functional profiling of microbial communities was performed using the PICRUSt2 pipeline [26], with pathway prediction based on Kyoto Encyclopedia of Genes and Genomes (KEGG) ortholog annotations.

### 2.8. Statistical Analysis

All statistical analyses were conducted using SPSS (version 23.0; IBM Corp., Armonk, NY, USA). One-way analysis of variance (ANOVA) was used to evaluate differences among treatment groups, followed by Duncan’s multiple range test. Comparisons between two independent groups were carried out using unpaired (independent-samples) *t*-tests. Because pens contained mixed sexes and complete individual sex information was not available for all endpoints, sex was not included as a fixed factor in the statistical models. Pens were balanced for sex to reduce potential systematic bias between dietary treatments. All data were expressed as mean ± standard error of the mean (SEM), and differences were considered statistically significant at *p* < 0.05. Graphs were prepared using GraphPad Prism (version 9.5; GraphPad Software, San Diego, CA, USA) and Adobe Illustrator CC 2018 (Adobe Systems Inc., San Jose, CA, USA).

## 3. Results

### 3.1. Growth Performance and Nutrient Digestibility

In comparison to the CON group, no statistically significant changes were observed in the FBW, ADG, ADFI, and F/G of weaned piglets in the LDC, MDC, and HDC groups (*p* > 0.05, Table 2).

Dietary supplementation with encapsulated CAPs had no significant effect on the digestibility of DM or CP in weaned piglets (*p* > 0.05, Table 3). In contrast, CF digestibility was significantly increased in the LDC and MDC groups compared with the CON group (*p* < 0.05).

### 3.2. Serum Biochemistry and Antioxidant Capacity

Dietary supplementation with encapsulated CAPs had no significant effects on serum TGs, T-Chol, HDL-C, T-AOC, SOD, or MDA in weaned piglets (*p* > 0.05, Table 4). In contrast, LDL-C was significantly decreased in the LDC, MDC, and HDC groups compared with CON (*p* < 0.05). Moreover, GSH-Px activity was markedly elevated in the MDC and HDC groups relative to CON and LDC (*p* < 0.05), indicating enhanced antioxidant capacity. These results suggest that encapsulated CAPs selectively modulate lipid metabolism and improve enzymatic antioxidant defense.

### 3.3. Organ Indices and Tissue Morphology

To investigate the effects on internal organs and tissues based on the established sampling rationale (see Section 2), piglets from the CON and LDC groups were selected and slaughtered for comparative analysis. There were no significant differences in organ indices of spleen, heart, kidney, and liver between the CON group and the LDC group in weaned piglets (*p* > 0.05, Table 5).

**Figure 1:**
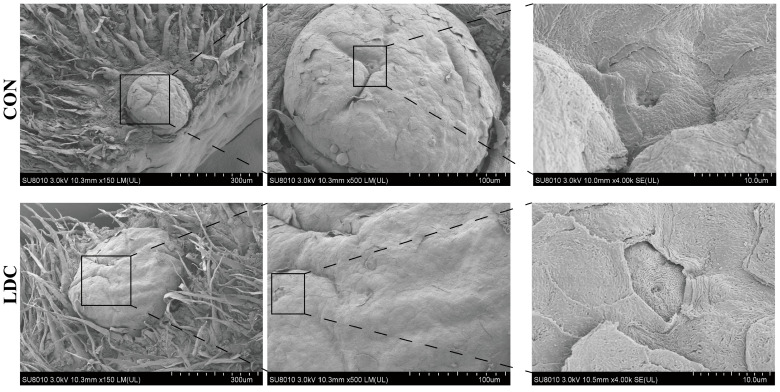
Effect of dietary supplementation with capsaicinoids on the development of taste buds in piglets. Scanning electron microscope of the fungiform papilla from CON and LDC. CON, basal diet provided as the control; LDC, basal diet supplemented with 200 mg/kg of an encapsulated capsaicinoids formulation.

Regarding the tongue taste buds, SEM revealed that dietary supplementation with encapsulated CAPs modulated tongue taste bud morphology in weaned piglets. Specifically, the LDC group exhibited enlarged fungiform papillae and shallower taste pores compared to the CON group (Figure 1).

**Figure 2:**
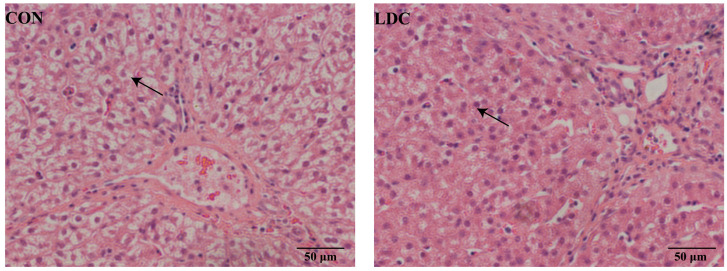
Effect of dietary supplementation with capsaicinoids on liver morphology in piglets (scale bars, 50 µm). Arrows indicate liver vacuolization. CON, basal diet provided as the control; LDC, basal diet supplemented with 200 mg/kg of an encapsulated capsaicinoids formulation.

For liver, H&E staining showed that compared with the CON group, the LDC group of weaned piglets exhibited a decreased degree of liver vacuolization (as shown by the arrow in Figure 2).

### 3.4. Intestinal Morphology and Tight Junction Protein Expression

**Figure 3:**
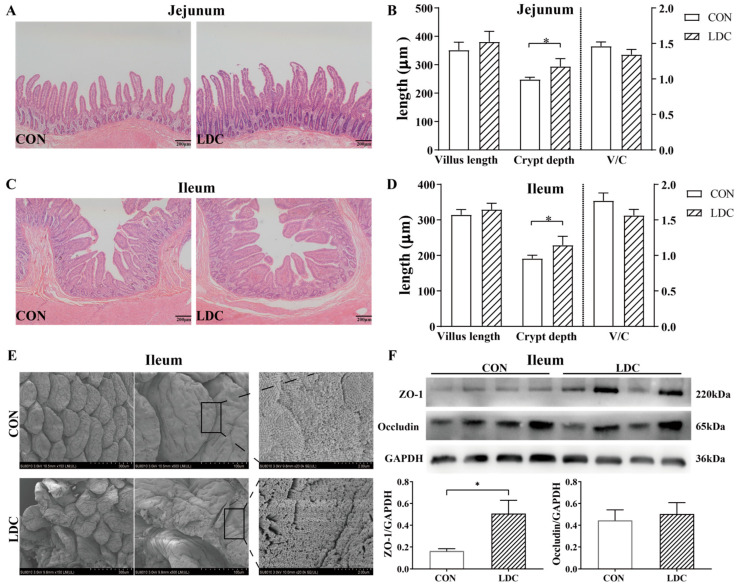
Effect of dietary supplementation with capsaicinoids on the intestinal morphology and tight junctional protein in piglets. (**A**) Representative H&E-stained jejunal sections and (**C**) ileal sections (scale bar = 200 µm). (**B**,**D**) Statistical analysis of villus height and crypt depth in the jejunum and ileum, respectively. (**E**) Scanning electron microscope of ileum mucosa (left, scale bar = 300 µm; middle, scale bar = 100 µm; right, scale bar = 2 µm). (**F**) Expression of the tight junction protein in ileum tissue. CON, basal diet provided as the control; LDC, basal diet supplemented with 200 mg/kg of an encapsulated capsaicinoids formulation. Bars represent mean ± SEM (n = 6). Star (*) indicates that there are significant differences between groups (*p* < 0.05).

No significant differences were observed between the LDC and CON groups in villus height or villus height-to-crypt depth (V/C) ratio in both the ileum and jejunum (*p* > 0.05, Figure 3A–D). Notably, the crypt depth in both the jejunum and ileum was significantly greater in the LDC group than in the CON group (*p* < 0.05). Figure 3E shows SEM of the ileal surface, where the LDC group exhibited more irregular and sparsely arranged microvilli compared with the CON group. Western blot analysis revealed significantly higher expression of ZO-1 in the ileum of the LDC group compared with the CON group (*p* < 0.05), whereas occludin expression did not differ significantly between the two groups (*p* > 0.05, Figure 3F).

### 3.5. Colonic Microbiota Composition

**Figure 4:**
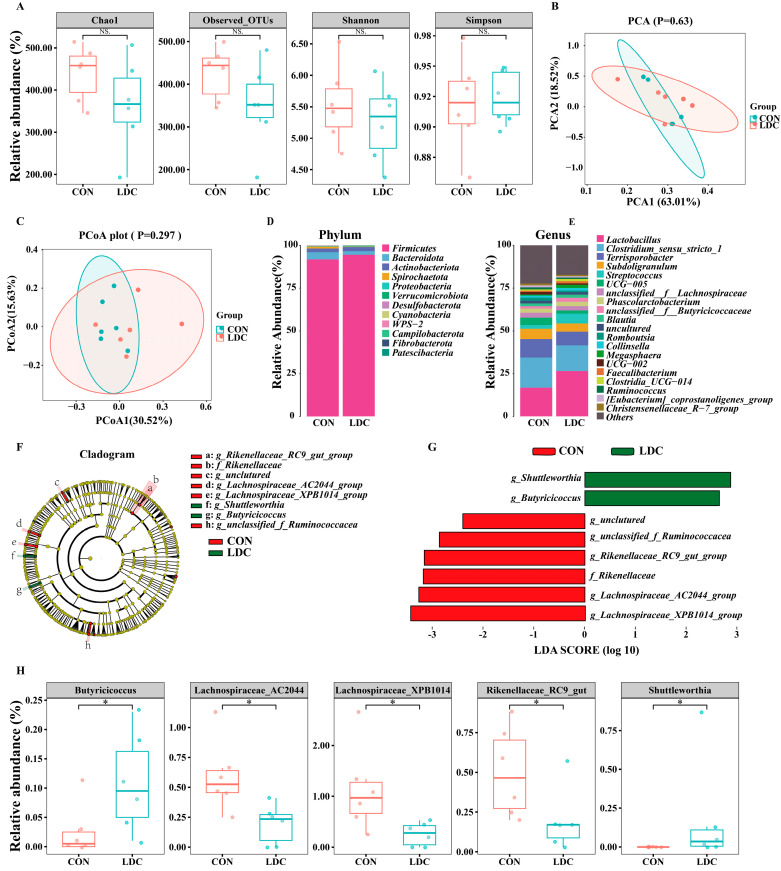
Effect of dietary supplementation with capsaicinoids on intestinal microbiota in piglets. (**A**) α diversity. (**B**) Principal Component Analysis. (**C**) Principal Co-ordinates Analysis. (**D**,**E**) Relative abundance of phylum level and genus level. (**F**,**G**) Cladogram and Histogram of LDA scores by LEfSe. (**H**) Differential bacteria in the colon of weaned piglets. CON, basal diet provided as the control; LDC, basal diet supplemented with 200 mg/kg of an encapsulated capsaicinoids formulation. The data was evaluated using the Wilcoxon rank-sum test (n = 6). Star (*) indicates that there are significantly different between groups; NS indicates no significant difference.

The results of 16S rDNA sequencing showed that there were no significant differences in the OTUs, Shannon, Chao1, and Simpson indices of colonic microbiota in weaned piglets between the CON and the LDC groups (*p* > 0.05) (Figure 4A). In terms of colonic microbiota β-diversity, PCA and PCoA analyses did not reveal distinct clustering between the CON and LDC groups (Figure 4B,C).

Although no significant alterations at the phylum level were observed (*p* > 0.05, Figure 4D), genus-level analysis revealed five differentially abundant taxa (Figure 4E). The cladogram highlighted eight key bacterial taxa (Figure 4F), and their differential abundance was quantified using LDA scores with a threshold of 2.0 (Figure 4G). Specifically, the LDC group showed a significantly lower relative abundance of *Lachnospiraceae_AC2044_group*, *Lachnospiraceae_XPB1014_group*, and *Rikenellaceae_RC9_gut* in the colonic microbiota of weaned piglets than the CON group. Conversely, the relative abundance of *Butyricicoccus* was significantly higher in the LDC group than in the CON group. Moreover, the genus *Shuttleworthia* was uniquely detected in the LDC group (*p* < 0.05, Figure 4H).

### 3.6. Predicted Microbial Functions

**Figure 5:**
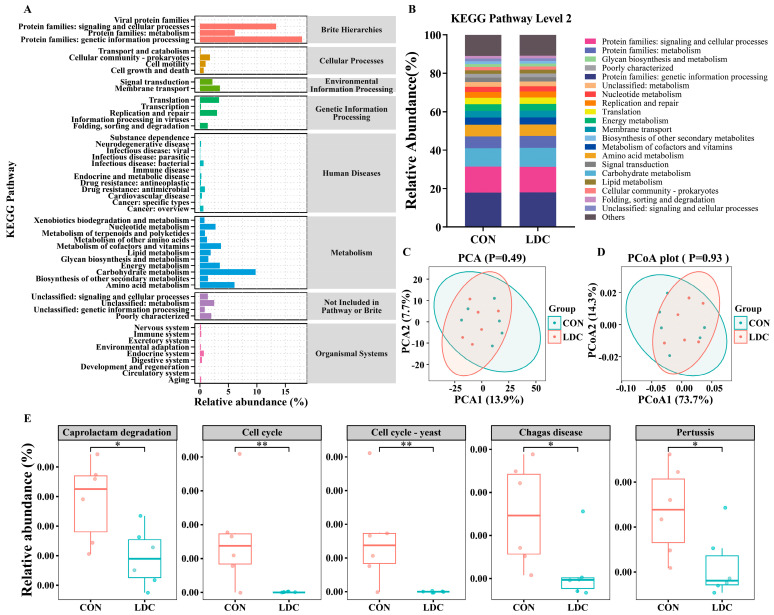
Functional analysis prediction. (**A**,**B**) KEGG pathway relative abundance. (**C**) Principal Component Analysis (Level 3). (**D**) Principal Co-ordinates Analysis (Level 3). (**E**) 5 KEGG pathway significantly differed between the CON and LDC (Level 3). CON, basal diet provided as the control; LDC, basal diet supplemented with 200 mg/kg of an encapsulated capsaicinoids formulation. The data was evaluated using the Wilcoxon rank-sum test (n = 6). Star (*) indicates that there are significant differences between groups (* *p* < 0.05, ** *p* < 0.01).

Figure 5A illustrates the relative abundances of metabolic pathways at Level 2, along with their associated Level 1 pathway classifications. Figure 5B presents the top 20 most abundant Level 2 metabolic pathways in the CON and LDC groups. At Level 3 resolution, although neither PCA nor PCoA revealed significant clustering patterns between groups (Figure 5C,D), five differentially abundant pathways were identified, including caprolactam degradation (ko00930), cell cycle (ko04110), cell cycle-yeast (ko04111), chagas disease (ko05142), and pertussis (ko05133) (Figure 5E). Moreover, all of these pathways exhibited significantly lower relative abundances in the LDC group compared to the CON group (*p* < 0.05, Figure 5E).

## 4. Discussion

In this study, dietary supplementation with encapsulated CAPs (0.47% CAP and 0.22% DHC) did not markedly improve short-term growth performance in weaned piglets. This outcome is consistent with the notion that early metabolic or digestive improvements do not necessarily translate into immediate growth responses during the post-weaning period. Nevertheless, encapsulated CAPs elicited measurable beneficial effects on crude fat digestibility, serum lipid status, antioxidant capacity, and intestinal morphology, with the LDC group showing the most consistent and pronounced responses. Based on these physiological findings, the CON and LDC groups were selected for slaughter sampling and mechanistic analyses. Taken together, these results suggest that encapsulated CAPs primarily influence early metabolic and physiological regulation, potentially through modified release kinetics in the gastrointestinal tract, rather than exerting an immediate impact on growth performance.

In terms of metabolic regulation, encapsulated CAPs exhibited a beneficial effect on lipid metabolism. In the present study, CAPs supplementation increased crude fat digestibility without significantly affecting DM or CP digestibility. This finding differs from that of Long, Liu, Wang, Mahfuz and Piao [21], who reported that free CAP enhanced DM and CP digestibility, suggesting that encapsulation alters the mode of action of CAPs, shifting its function from a general digestive stimulant to a specific lipid metabolism regulator. Serum biochemical analysis further supported this observation: the LDC group showed a significant reduction in serum LDL-C, a key indicator responsible for transporting cholesterol to extrahepatic tissues and closely associated with lipid deposition [27]. This result is consistent with the effect observed in the rat model reported by Zhang, et al. [28]. The improvement in lipid metabolism may involve multiple interconnected mechanisms. On one hand, CAPs activate TRPV1 receptors, upregulating the expression of genes related to fatty acid oxidation and mitochondrial respiration [29], thereby systemically enhancing lipid catabolism. On the other hand, CAPs can modulate gut microbiota composition, alter short-chain fatty acid concentrations, and indirectly regulate lipid homeostasis [30], as later confirmed by the observed enrichment of butyrate-producing bacteria. Notably, histological examination of liver tissue revealed reduced hepatocellular vacuolation in the LDC group, providing morphological evidence for improved hepatic lipid metabolism. Taken together, these results suggest that encapsulated CAPs establish a metabolic regulatory pathway that enhances intestinal fat digestion efficiency, promotes LDL-C clearance from circulation, and alleviates hepatic lipid accumulation. This mechanism not only explains the functional difference between encapsulated and free CAPs but also highlights the potential of encapsulated formulations for precise regulation of lipid metabolism.

Regarding oxidative homeostasis, the endogenous antioxidant system in animals generally maintains balance under normal physiological conditions, but can be disrupted under stress, leading to oxidative stress that impairs growth and health [31]. CAPs have been demonstrated to possess strong antioxidant potential in models of oxidative stress induced by LPS or high-fat diets [32,33]. In this study, dietary CAPs significantly increased serum GSH-Px activity in weaned piglets, whereas total antioxidant T-AOC, SOD, and MDA levels remained unchanged, suggesting that its antioxidant effect mainly depends on enhanced GSH-Px activity. This finding is consistent with the effects observed in laying ducks by Liu, et al. [34]. As a key glutathione-dependent enzyme, GSH-Px reduces hydrogen peroxide and organic peroxides to maintain redox balance, protect tissues from oxidative damage, and support immune function [35]. Collectively, these results indicate that CAPs selectively strengthen the antioxidant defense system in weaned piglets, acting synergistically with enhanced lipid digestibility and reduced serum LDL-C levels to support lipid metabolic homeostasis and energy utilization efficiency.

The intestine is the main site of nutrient absorption in animals, and its structural integrity is essential for maintaining normal digestive and absorptive functions. Intestinal crypts continuously generate new epithelial cells, reflecting the proliferative and differentiative capacity of the epithelium [36]. Histological analysis in this study revealed that CAPs promoted adaptive intestinal remodeling in weaned piglets: the jejunal and ileal crypts in the LDC group were significantly deeper, indicating active epithelial renewal that may help mitigate weaning stress–induced mucosal damage. The improvement in crude fat digestibility further supports the contribution of this structural remodeling to nutrient absorption efficiency. In terms of barrier function, CAPs significantly upregulated the expression of the tight junction protein ZO-1 in the ileum, consistent with in vitro findings reported by Zhao, et al. [37]. ZO-1 plays a pivotal role in maintaining epithelial integrity and regulating barrier function during cellular stress responses [38]. The coordinated changes of crypt deepening and ZO-1 upregulation suggest that CAPs may improve intestinal barrier function through dual mechanisms—enhancing epithelial renewal and reinforcing cell–cell junctions. It is noteworthy that while deepened crypts are traditionally associated with impaired absorptive function, the concurrent increase in fat digestibility and ZO-1 expression in this study indicates adaptive repair rather than pathological alteration following weaning [39]. Moreover, scanning electron microscopy revealed slight villus tip damage in the LDC group, suggesting that although CAPs promote epithelial renewal, localized high concentrations released in situ may cause mild mucosal irritation. This finding underscores the need for further optimization of release kinetics to balance efficacy and safety. Overall, encapsulated CAPs improve intestinal health in weaned piglets by promoting epithelial regeneration and enhancing tight junction integrity, though refinement of its sustained-release profile remains necessary for optimal outcomes.

In mammals, taste buds are primarily located on the fungiform, foliate, and circumvallate papillae of the tongue, and their development can be influenced by early taste experiences, which affect feeding behavior [40]. In this study, weaned piglets fed encapsulated CAPs exhibited enlarged fungiform papillae and shallower taste pores, indicating the presence of bioactive compounds in the oral cavity. This likely resulted from minimal early release of CAPs from the microcapsules, sufficient to stimulate highly sensitive TRP receptors on taste buds or indirectly via systemic signaling following intestinal absorption [41,42]. Notably, feed intake was unaffected, suggesting that oral exposure was below the threshold for aversive responses, and the morphological changes reflect local physiological adaptations rather than behavioral rejection. These results support the encapsulation strategy’s efficacy in reducing acute oral pungency while delivering CAPs to the intestine, contributing to improved intestinal health and metabolic function without compromising palatability.

Microbiota analysis further elucidated the potential mechanisms underlying these effects. Previous studies have demonstrated that CAPs exhibit potent antimicrobial effects both in vivo and in vitro [43], protect mice against bacterial infection in vivo [44], and remodel gut microbial communities [45]. In this study, the colonic microbiota of both CON and LDC groups was dominated by Firmicutes and Bacteroidetes, with Firmicutes accounting for more than 90% of the total abundance, consistent with previous observations [46]. Alpha and beta diversity analyses indicated that the overall community structure remained stable. However, at the genus level, CAPs induced selective modulation: the relative abundances of *Lachnospiraceae_AC2044_group*, *Lachnospiraceae_XPB1014_group*, and *Rikenellaceae* were significantly decreased. The first two belong to the *Lachnospiraceae* family under Firmicutes; although *Lachnospiraceae_AC2044_group* is known as a butyrate producer involved in dietary fermentation in ruminants, studies in pigs are limited [47,48]. *Rikenellaceae* comprises anaerobic hydrogen-producing bacteria that ferment carbohydrates to produce acetate, hydrogen, carbon dioxide, and hydrogen sulfide, potentially exerting adverse effects on host health [49]. In addition, CAPs supplementation increased the relative abundance of the butyrate-producing genus *Butyricicoccus*, which may elevate colonic butyrate levels, optimize microbial structure, and improve intestinal absorptive function [50]. Interestingly, the genus *Shuttleworthia* was detected only in the LDC group, and although its specific role in animal health and metabolism remains unclear. Notably, the increase in *Butyricicoccus* paralleled improvements in ileal ZO-1 and fat digestibility. Since butyrate strengthens the intestinal barrier and modulates lipid homeostasis, our findings support a plausible mechanism whereby CAPs enrich butyrate producing taxa to promote host health. While SCFA levels were not quantified, the synchronized microbial and phenotypic changes indicate a functional reshaping of gut microbiota.

Based on 16S rRNA sequencing data, PICRUSt2 was employed to predict microbial functional profiles and compare metabolic pathways between the CON and LDC groups [51]. No significant differences were observed at KEGG levels 1 and 2, and PCA/PCoA analyses at level 3 showed no distinct clustering, suggesting that overall functional characteristics remained stable. However, KEGG-based pathway analysis revealed consistent downregulation of certain pathways in the LDC group, including caprolactam degradation, cell cycle control, and host–pathogen interaction pathways such as Chagas disease and pertussis. These findings indicate that CAPs supplementation may suppress excessive microbial proliferation, reduce the metabolic burden of exogenous compounds, and modulate local immune responses, thereby creating a favorable intestinal microenvironment. Together, these microbial changes, characterized by increased butyrate-producing bacteria and selective regulation of functional taxa, are likely linked to improvements in lipid metabolism, intestinal barrier integrity, and hepatic lipid accumulation. Overall, encapsulated CAPs maintain gut microbial homeostasis while promoting microbe–host interactions that optimize lipid utilization, intestinal structure, and local immune environment in weaned piglets, thereby supporting long-term metabolic balance and physiological health.

Despite the valuable insights gained from this study, several limitations should be noted. First, incomplete sex metadata precluded a full diet-by-sex analysis, and sex-related variation, particularly in gut microbiota, may have contributed to variability [52]. Second, microbial fermentation products such as SCFAs, phenolic compounds, and ammonia were not quantified because appropriate digesta samples were not collected. This limits the functional interpretation of the observed microbial shifts and intestinal responses. Third, mechanistic analyses of intestinal morphology, tight junction proteins, and colonic microbiota were conducted only in the CON and LDC groups, whereas a full dose comparison would provide clearer insight into dose-dependent effects. Finally, although encapsulated CAPs improved lipid metabolism, antioxidant status, and nutrient digestion, short-term growth performance remained unchanged, likely due to the brief trial period. Tissue-level lipid analyses, including lipidomics, were also not performed.

Future studies should span the entire fattening period, include sex as an independent factor, collect digesta suitable for metabolite profiling, and incorporate multi-dose tissue analyses to better elucidate the mechanisms of encapsulated CAPs.

## 5. Conclusions

Although encapsulated CAPs did not exert a significant effect on the growth performance of weaned piglets, they demonstrated multiple beneficial effects in improving fat digestibility, modulating lipid metabolism, and enhancing antioxidant capacity. Notably, supplementation with a low dose of the encapsulated product (200 mg/kg, equivalent to 1.38 mg/kg pureCAPs) exhibited the most pronounced positive outcomes. This dosage not only enhanced antioxidant activity and improved lipid metabolism but also promoted intestinal development, stimulated taste bud maturation, alleviated hepatic vacuolation, and modulated the composition of the colonic microbiota. These findings suggest that encapsulated CAPs hold potential as a functional additive for regulating the health of weaned piglets. Overall, the encapsulated formulation may shift the biological activity of CAPs from a short-term digestive stimulant to a long-term metabolic regulator, highlighting its potential as a functional feed additive for maintaining post-weaning health.

## Figures and Tables

**Table 1:** Basic composition of diet and nutritional level.

Ingredients, %		Nutritional Level ^a^	
Corn	43.40	Digestible energy kcal/kg	3444
Puffed corn	16.00	Metabolisable energy, kcal/kg	3173
Soybean meal	10.00	Net energy, kcal/kg	2510
Expanded soybean	10.00	Crude protein (%)	19.04
Fermented soybean meal	5.60	Crude fat (%)	5.40
Fish meal	3.00	Crude fiber (%)	2.03
Whey powder	3.00	Calcium (%)	1.02
Glucose	2.00	Phosphorus (%)	0.59
Egg yolk powder	1.00	Sodium (%)	0.26
Soybean oil	1.00	Chlorine (%)	0.33
Stone powder	1.00	Salinity (%)	0.57
Calcium bicarbonate	0.90	SID ^c^ lysine (%)	1.18
Wheat flour	0.59	SID methionine (%)	0.36
NaCl	0.35	SID threonine (%)	0.77
L-Lysine hydrochloride (98.5%)	0.30		
Acidifier	0.30		
Mineral agent	0.20		
L-Threonine (98.5%)	0.15		
Choline chloride (50%)	0.10		
DL-Methionine (98.5%)	0.06		
Fungal adsorbent	0.05		
Premix ^b^	1.00		
Total	100		

^a^ Digestive energy, metabolic energy and net energy are calculated values, whereas the levels of other nutrients were analyzed. ^b^ Provided per kg of diet: Vitamin A, 12,000 IU; Vitamin D_3_, 2700 IU; Vitamin E, 75 mg; Vitamin K, 1.25 mg; Vitamin B_1_, 1.5 mg; Vitamin B_2_, 2.5 mg; Vitamin B_6_, 2.5 mg; Vitamin B_12_, 0.04 mg; Niacin, 34.4 mg; Mn, 50 mg; Cu, 100 mg; Fe, 110 mg; Se, 0.5 mg; Zn, 100 mg; I, 0.5 mg. ^c^ Standardized ileal digestible.

**Table 2:** Effect of dietary supplementation with capsaicinoids on growth performance in piglets.

Items	CON	LDC	MDC	HDC	SEM	*p*-Value
IBW, kg	7.56	7.56	7.60	7.61	0.057	0.980
FBW, kg	20.70	21.01	20.71	20.78	0.296	0.981
ADG, g/d	375.7	384.3	374.3	374.3	9.72	0.983
ADFI, g/d	667.1	678.6	662.9	655.7	15.65	0.968
F/G	1.78	1.77	1.77	1.75	0.013	0.863

Abbreviations: CON, basal diet provided as the control; LDC, basal diet supplemented with 200 mg/kg of an encapsulated capsaicinoids formulation; MDC, with 400 mg/kg; HDC, with 600 mg/kg; IBW, initial body weight; FBW, final body weight; ADG, average daily gain; ADFI, average daily feed intake; F/G, feed-to-gain ratio.

**Table 3:** Effect of dietary supplementation with capsaicinoids on digestibility of nutrients in piglets.

Items	CON	LDC	MDC	HDC	SEM	*p*-Value
Dry matter, %	85.88	86.19	86.70	85.97	0.361	0.879
Crude fat, %	71.80 ^b^	80.63 ^a^	81.75 ^a^	77.18 ^a,b^	1.170	0.009
Crude protein, %	79.22	79.97	80.24	82.32	0.701	0.521

Abbreviations: CON, basal diet provided as the control; LDC, basal diet supplemented with 200 mg/kg of an encapsulated capsaicinoids formulation; MDC, with 400 mg/kg; HDC, with 600 mg/kg. ^a,b^ Means within a row with different letters differed significantly (*p* < 0.05).

**Table 4:** Effect of dietary supplementation with capsaicinoids on serum biochemical indices and antioxidant capacity in piglets.

Items	CON	LDC	MDC	HDC	SEM	*p*-Value
TGs, mmol/L	0.79	0.63	0.55	0.67	0.042	0.213
T-Chol, mmol/L	3.28	3.25	3.06	2.92	0.106	0.606
LDC-C, mmol/L	1.57 ^a^	1.25 ^b^	1.23 ^b^	0.99 ^b^	0.059	0.004
HDL-C, mmol/L	1.27	1.32	1.20	1.25	0.044	0.782
T-AOC, µmol/L	195.9	199.2	181.7	211.0	9.831	0.785
SOD, U/mL	32.60	36.72	37.12	34.09	1.572	0.716
GSH-Px, U/mL	590.5 ^b^	637.9 ^b^	774.4 ^a^	793.4 ^a^	20.26	<0.001
MDA, nmol/mL	2.65	3.11	2.53	2.64	0.100	0.189

Abbreviations: CON, basal diet provided as the control; LDC, basal diet supplemented with 200 mg/kg of an encapsulated capsaicinoids formulation; MDC, with 400 mg/kg; HDC, with 600 mg/kg; TGs, triglycerides; T-Chol, total cholesterol; LDL-C, low-density lipoprotein cholesterol; HDL-C, high-density lipoprotein cholesterol; T-AOC, total antioxidant capacity; SOD, superoxide dismutase; GSH-Px, glutathione peroxidase; MDA, malondialdehyde. ^a,b^ Means within a row with different letters differed significantly (*p* < 0.05).

**Table 5:** Effect of dietary supplementation with capsaicinoids on organ indices in piglets.

Items	CON	LDC	SEM	*p*-Value
Absolute weight of organs, g				
Spleen	54.42	55.73	1.978	0.756
Heart	94.68	95.57	1.283	0.748
Kidney	110.2	102.7	3.442	0.303
Liver	628.1	644.7	22.507	0.731
Relative weight of organs, g/kg				
Spleen	2.63	2.69	0.085	0.739
Heart	4.61	4.60	0.087	0.937
Kidney	5.37	4.96	0.216	0.362
Liver	30.38	30.99	0.853	0.738

Abbreviations: CON, basal diet provided as the control; LDC, basal diet supplemented with 200 mg/kg of an encapsulated capsaicinoids formulation.

## Data Availability

The original contributions presented in this study are included in the article. Further inquiries can be directed to the corresponding author.

## Abbreviations

The following abbreviations are used in this manuscript:

CAPs	Capsaicinoids
TRPV1	Transient receptor potential vanilloid 1
CAP	Capsaicin
DHC	Dihydrocapsaicin
ADFI	Average daily feed intake
ADG	Average daily gain
F/G	Feed-to-gain ratio
IBW	Initial body weight
FBW	Final body weight
DM	Dry matter
CP	Crude protein
CF	Crude fat
AIA	Acid-insoluble ash
ATTD	Apparent total tract digestibility
TGs	Triglycerides
T-Chol	Total cholesterol
LDL-C	Low-density lipoprotein cholesterol
HDL-C	High-density lipoprotein cholesterol
T-AOC	Total antioxidant capacity
SOD	Superoxide dismutase
GSH-Px	Glutathione peroxidase
MDA	Malondialdehyde
ZO-1	Zonula occludens-1
PCoA	Principal coordinates analysis
PCA	Principal component analysis
KEGG	Kyoto Encyclopedia of Genes and Genomes
